# Global research trends at the intersection of climate change and parasitic diseases: a systematic bibliometric research (2000–2025)

**DOI:** 10.3389/fcimb.2026.1804158

**Published:** 2026-05-04

**Authors:** Maireyamuguli Nuermaimaiti, Zile Liu, Yimei Xu, Xinwei Qi

**Affiliations:** 1 Xinjiang Medical University First Affiliated Hospital Medical Laboratory Center, Urumqi, China; 2 School of Public Health, Xinjiang Medical University, Urumqi, China; 3 College of Anesthesiology, Southern Medical University, Guangzhou, China; 4 The First Affiliated Medical College, Southern Medical University, Guangzhou, China; 5 Xinjiang Uyghur Autonomous Region Center for Disease Control and Prevention, Urumqi, China

**Keywords:** bibliometric studies, climate change, one health, parasitic diseases, visual analysis

## Abstract

**Background:**

Climate change is increasingly recognized as a critical driver affecting the transmission and distribution of parasitic diseases. However, a comprehensive synthesis of the global research landscape in this interdisciplinary field is lacking. This study provides an integrated analysis of research trends, collaborative networks, and emerging themes from 2000 to 2025.

**Methods:**

We retrieved relevant articles and reviews from Web of Science and Scopus, analyzing 7,303 publications using visualization and statistical tools. Bibliometric analysis included publication trends, contributions by countries/institutions, author networks, journal profiles, and keyword evolution.

**Results:**

Annual publications grew steadily at 13.1%. The United States led in output (5,085 papers) and citations (55,533), with strong international collaboration (40.5% cooperation rate). Key journals included *Veterinary Parasitology* and high-impact journals such as *Science* and *Nature*. Authors clustered into five major collaborative groups, with Poulin R. and Johnson P. T. J. as the most productive and influential researchers, respectively. Keyword analysis identified core themes including zoonoses, climate epidemiology, One Health, and specific diseases like malaria and schistosomiasis. Emerging keywords such as “One Health” and “Surveillance” showed annual growth exceeding 200%.

**Conclusion:**

By integrating advanced bibliometric analysis, this study provides new insights. Specifically, research on climate change and parasitic diseases is evolving toward interdisciplinary and systems-oriented approaches. Future efforts should prioritize predictive modeling, global health governance, and integrating biodiversity conservation with disease control to mitigate climate-related health risks.

## 1 Introduction

Global climate change has emerged as one of the most pressing environmental and health challenges of the 21st century. Its extensive impacts extend not only to ecosystem structure and function but also directly threaten human health, particularly by altering the transmission dynamics of infectious diseases, the geographical ranges of vectors, and the life cycles of pathogens—thereby significantly influencing the epidemiology and control of parasitic diseases (Pfenning-Butterworth et al., 2024). Parasitic diseases, which are closely associated with poverty, sanitation conditions, and ecological environments, impose a substantial health burden worldwide, especially in tropical and subtropical regions (Zhu et al., 2024). Climate change, through mechanisms such as rising temperatures, shifting precipitation patterns, and the increasing frequency of extreme weather events, can directly or indirectly affect the geographic distribution, population dynamics, and transmission intensity of parasites and their vectors (Kargbo et al., 2025). For example, the larval stages of lung flukes and hookworms require development within specific intermediate hosts—organisms living in aquatic or soil environments—whose reproduction and survival are influenced by humidity, thereby increasing the transmission risk of such parasitic diseases (Quin et al., 2012; Yoshida et al., 2019). Beyond gradual changes in mean temperature and precipitation, climate variability—such as seasonal fluctuations and phenomena like El Niño—can drive interannual fluctuations in disease incidence. Extreme climate events, including floods, droughts, and heatwaves, may have more immediate and profound impacts. Floods can expand the breeding grounds for vectors and intermediate hosts (e.g., snails for schistosomiasis), while droughts can concentrate water sources, increasing human–vector contact. Heatwaves can accelerate parasite development within vectors and compromise host immune responses. Furthermore, prolonged high temperatures can elevate the average temperature of water bodies, degrade water quality, or trigger flooding, which may accelerate pathogen proliferation and exacerbate the disease burden associated with waterborne zoonotic infections.

In the field of clinical medicine, detailed bibliometric analyses have been conducted on topics such as COVID-19 (Yu et al., 2020), Parkinson’s disease (Abumalloh et al., 2024), perioperative hypotension (Wang et al., 2023), and bone metastases in prostate cancer (Liu et al., 2025). Within the realm of climate change and health research, numerous scholars have explored the relationship between climatic variables and the transmission of parasitic diseases from various perspectives. Some studies have used predictive models to demonstrate the enhancing effect of rising temperatures on the transmission potential of malaria (Megersa and Luo, 2025), while others have focused on the impact of precipitation changes on the distribution of snail intermediate hosts in schistosomiasis (Nkolokosa et al., 2025). However, most of these investigations concentrate on single diseases or specific regions, lacking a systematic synthesis and knowledge-structural analysis of the broader research domain. Bibliometrics, as a research tool grounded in mathematical and statistical methods for analyzing academic literature, can systematically reveal publication trends, knowledge structures, collaboration networks, and research frontiers within a given field (Blumrich et al., 2025). In the area of climate change and infectious diseases, several bibliometric studies have addressed malaria (Klepac et al., 2024) or vector-borne diseases in a broader sense (Pham et al., 2026). Nevertheless, systematic bibliometric analyses specifically targeting the more extensive and heterogeneous theme of climate change and parasitic diseases remain scarce. Existing reviews tend to emphasize specific diseases or mechanistic discussions, often failing to provide a comprehensive overview of the overall development trends, distribution of research capacity, and evolution of knowledge in this interdisciplinary field from a macroscopic knowledge-mapping perspective (Caro, 2023). Given the inextricable links between human health, animal health, and ecosystem integrity in the context of climate change, this study adopts an inclusive interpretation of ‘parasitic diseases.’ It encompasses not only clinical diseases in humans but also the broader parasitology literature in veterinary, wildlife, and ecological sciences, reflecting the holistic perspective of the One Health paradigm. Therefore, to address this research gap, this study aims to conduct a comprehensive and systematic bibliometric analysis of the scientific literature on climate change and parasitic diseases. By integrating relevant publications from the core databases of Web of Science (WOS) and Scopus and utilizing visualization and analytical tools such as VOSviewer, CiteSpace, and Bibliometrix, this research will focus on addressing the following key questions: (1) What are the annual publication trends, and what are the contribution patterns of core countries/regions and research institutions in this field? (2) What are the characteristics of key authors, highly influential publications, and collaboration networks? (3) What are the main research themes, the evolution of hotspots, and future frontier directions? The findings aim to provide a more intuitive, comprehensive, and systematic reference for clinical practitioners and researchers in related fields.

## 2 Materials and methods

### 2.1 Data sources and search strategy

The data for this study were sourced from two authoritative databases: Scopus and WOS Core Collection. The search strategy was as follows: in WOS, the “Title” field was used as the search scope; in Scopus, the “Title-Abstract-Keywords” field was used. The search terms were “(parasitic disease* OR parasitic infection* OR Parasitosis OR parasite) AND (climate OR climate variability OR meteorological* OR meteorologic* OR meteorologically OR climate change OR weather)”. Literature types were limited to articles (research papers) or reviews, excluding conference abstracts, editorial materials, letters, retracted publications, and duplicate publications. No language restrictions were applied. The time span covered 2000–2025 (note: data for 2025 were incomplete). All searches and downloads were completed on January 14,2026.

We acknowledge the inherent asymmetry in search fields between the two databases (Title in WOS vs. TITLE-ABS-KEY in Scopus). This approach was deliberately chosen to balance precision and recall. To assess potential bias, we conducted a *post-hoc* sensitivity check on a random sample of 100 recent publications, which confirmed that the core thematic representation of the retrieved corpus was not systematically distorted by this methodological choice. Researchers should, however, consider this nuance when interpreting the results. All keyword analyses were performed on a merged dataset combining both author keywords and indexed keywords. This approach balances author-specific terminology with standardized indexing terms, providing a more comprehensive representation of the thematic landscape.

### 2.2 Data processing and visualized analysis

The “complete records and cited references” option was selected to export the literature in plain text format. The relevant data from the included literature were imported into CiteSpace, VOSviewer, Excel 365, and Bibliometrix for bibliometric and visual analysis. VOSviewer was used to construct and visualize collaboration networks among countries, institutions, and authors, as well as co-citation and keyword co-occurrence networks. CiteSpace was applied to perform keyword clustering and journal dual-map overlay analysis to reveal research hotspots and frontier trends. The Bibliometrix package was utilized to extract important information such as documents, authors, sources, and keywords from the literature dataset, and scientific plotting was conducted using the ggplot2 package. To ensure accuracy and consistency in data processing, VOSviewer’s built-in thesaurus was employed to standardize country names, author names, and closely related keywords. Manual verification was then conducted. To ensure data quality, we performed an iterative cleaning process. An initial merge was conducted using VOSviewer’s thesaurus. Subsequently, all keywords with a frequency more than 5 were manually reviewed and standardized by two researchers to correct misspellings, merge synonyms, and resolve inconsistencies. The final controlled vocabulary used for all keyword-based analyses is provided in **Supplementary Material**. This merged set was used for all co−occurrence, clustering, and trend analyses, as it balances the author−specific terminology with standardized indexing terms, thereby providing a more complete representation of the research content. For country and institution-level analyses, we employed full counting, a standard bibliometric method where a publication is credited to each affiliated country or institution. Therefore, the sum of country-level publications exceeds the total number of documents in the dataset. Keyword correlation analysis was based on the co-occurrence matrix generated by Bibliometrix. All data screening was cross-checked by two researchers to ensure the rigor of this study.

Annual growth rate was calculated as the compound annual growth rate (CAGR) of publication counts over the study period. International collaboration rate was defined as the proportion of publications with authors from at least two different countries, relative to all publications. This was computed based on the affiliation information of all co−authors. Cooperation rate (used interchangeably with international collaboration rate in this study) follows the same definition. Keyword growth trends were evaluated using linear regression of annual frequency counts. Instead, keywords were ranked by both total frequency and growth slope to identify emerging topics.

**Figure 1:**
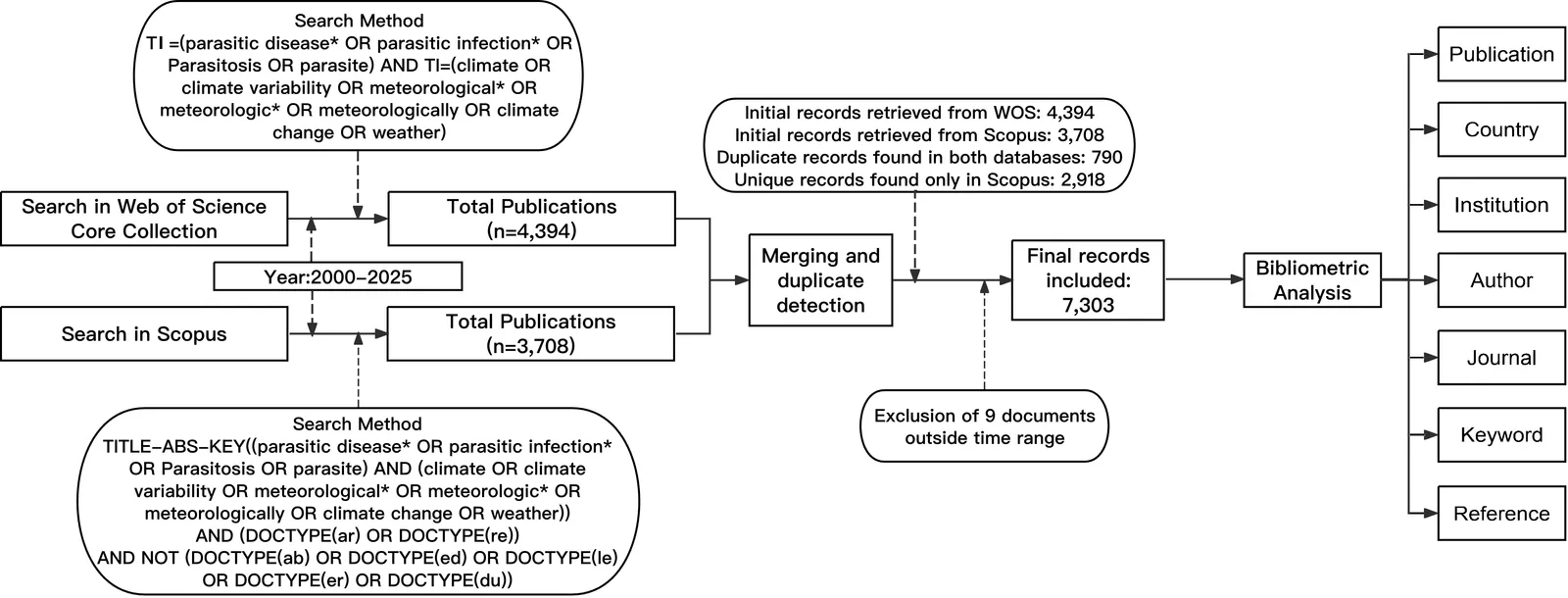
Technical roadmap for data filtering and cleaning.

**Figure 1** illustrates the process of data identification and selection strategies in this study.

## 3 Results

**Table 1** presents the results of descriptive analysis performed on the aforementioned literature data using the Bibliometrix package in R software. The 7,303 publications included in this study (Articles: 6,045; Reviews: 1,258) were contributed by 33,078 authors and published in 1,668 journals. In terms of research collaboration characteristics, this field exhibits a high degree of cooperation. The average number of co-authors per publication is 5.65, and the international collaboration rate reaches 40.5%, indicating a pronounced pattern of international cooperation. Single-author publications account for only 5.4% (392 articles), meaning the vast majority of studies are conducted collaboratively. The mean publication age is 7.89 years, and the average number of citations per publication is as high as 29.66, suggesting that research outputs in this domain maintain sustained influence. Additionally, the use of a total of 24,843 indexed keywords and 16,454 author keywords reflects the diversity and richness of research topics.

**Table 1:** Descriptive analysis results of the literature on climate change and parasitic diseases.

Description	Results	Description	Results
Timespan	2000-2025	Sources(Journals, Books, etc)	1668
Annual Growth Rate%	13.1	Authors	33078
Documents	7303	Authors of single-authored docs	365
Document Average Age	7.89	Single-authored docs	392
Article	6045	Co-Authors per doc	5.65
Review	1258	International co-authorships%	40.5
Keywords Plus	24843	Average citations per doc	29.66
Author’s Keywords	16454	References	241558

### 3.1 General trend

**Figure 2:**
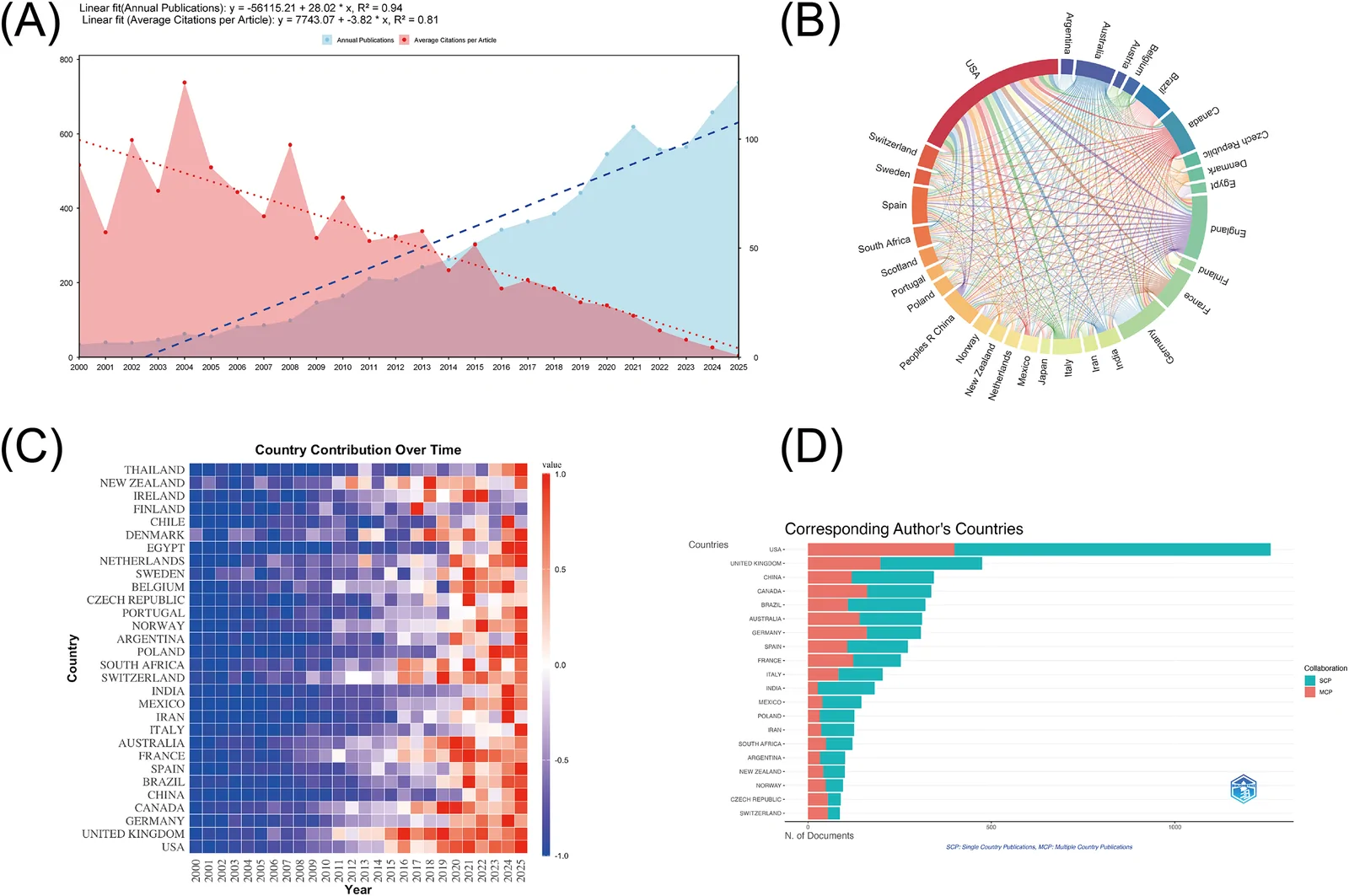
**(A)** Description of publication volume and citation frequency. From 2000 to 2025, the annual number of publications in the field of climate and parasitic diseases showed a steady increase (linear fit equation y=-56115.21 + 28.02*x,R² = 0.94), while the average number of citations per article exhibited a gradual declining trend (linear fit equation=7743.07-3.68*x,R²=0.81). **(B)** Distribution and international collaboration network of research on climate change and parasitic diseases. The thickness of the lines represents the strength of collaboration; thicker lines indicate stronger collaborative relationships**. (C)** Heatmap of the contribution and activity of countries participating in research related to climate change and parasitic diseases. Color intensity reflects the level of research engagement of each country in different years. **(D)** Paper output by country of the corresponding author in the field of climate change and parasitic disease research. Bar length represents the number of publications from each country. SCP denotes single-country/region publications, and MCP denotes multiple-country/region publications.

The volume of published literature serves as an important indicator for measuring development trends and emerging hotspots in a research field, while citation counts effectively reflect the level of scholarly exchange and activity within the domain. **Figure 2A** shows that the annual publication output increased from 34 articles in 2000 to 738 articles in 2025. Linear fitting results indicate a stable growth trend in publication volume (R²=0.94), with an average annual growth rate reaching 13.1%, particularly accelerating noticeably after 2015. However, alongside the continuous growth in research output, the average number of citations per article shows a declining trend (linear fitting R²=0.81), decreasing from over 40 citations per article in earlier years to less than 20 citations in recent years. This may reflect the rapid expansion of research scale and the fact that newly published literature has not yet reached its peak citation period.

### 3.2 Distributions of countries/regions

A total of 151 countries/regions have conducted research related to climate and parasitic diseases, with their geographical distribution concentrated in North America. The United States had the highest level of research participation, being affiliated with 5,085 papers, which accounted for its involvement in 69.6% of all publications based on full counting, with a total of 55,533 citations, far exceeding those of other countries. The United Kingdom ranks second with 1,650 publications and 24,725 total citations, demonstrating significant influence in this field. Germany, Canada, China, Brazil, Spain, France, Australia, and Italy occupy the third to tenth positions, respectively, forming the main cohort of research contributors (**Table 2**).

**Table 2:** Top10 countries/regions related to climate and parasitic diseases.

Rank	Country/regions	Documents	Rank	Country/regions	Total citations
1	USA	5085	1	USA	55533
2	UK	1650	2	UK	24725
3	Germany	1184	3	Australia	11978
4	Canada	1159	4	Canada	11885
5	China	1148	5	Spain	8759
6	Brazil	1142	6	France	7931
7	Spain	1035	7	Germany	6640
8	France	1004	8	New Zealand	5940
9	Australia	997	9	Italy	5926
10	Italy	780	10	China	5666

The international collaboration network analysis (**Figure 2B**) reveals the cooperative patterns among countries. The thickness of the connecting lines in the figure represents the strength of collaboration, indicating that the United States has established the closest partnerships with countries such as the United Kingdom, Canada, Germany, and Australia. Collaboration within Europe (e.g., between the UK and Germany, and between the UK and France) is also relatively active. Among Asian countries, China has developed strong collaborative relationships with the United States, the United Kingdom, Australia, and others. The heatmap (**Figure 2C**) further illustrates the changing contributions of major research countries across different years. It shows that the United States maintained high output across all years, while developing countries such as China have seen continuously increasing research activity in recent years. Corresponding author analysis (**Figure 2D**) offers another perspective, indicating that the United States has the highest number of publications with corresponding authors, while Germany leads in the proportion of internationally co-authored publications (MCP), and India produces a higher share of single-country publications (SCP), reflecting differences in research patterns among countries.

### 3.3 Distribution by institutions

**Figure 3:**
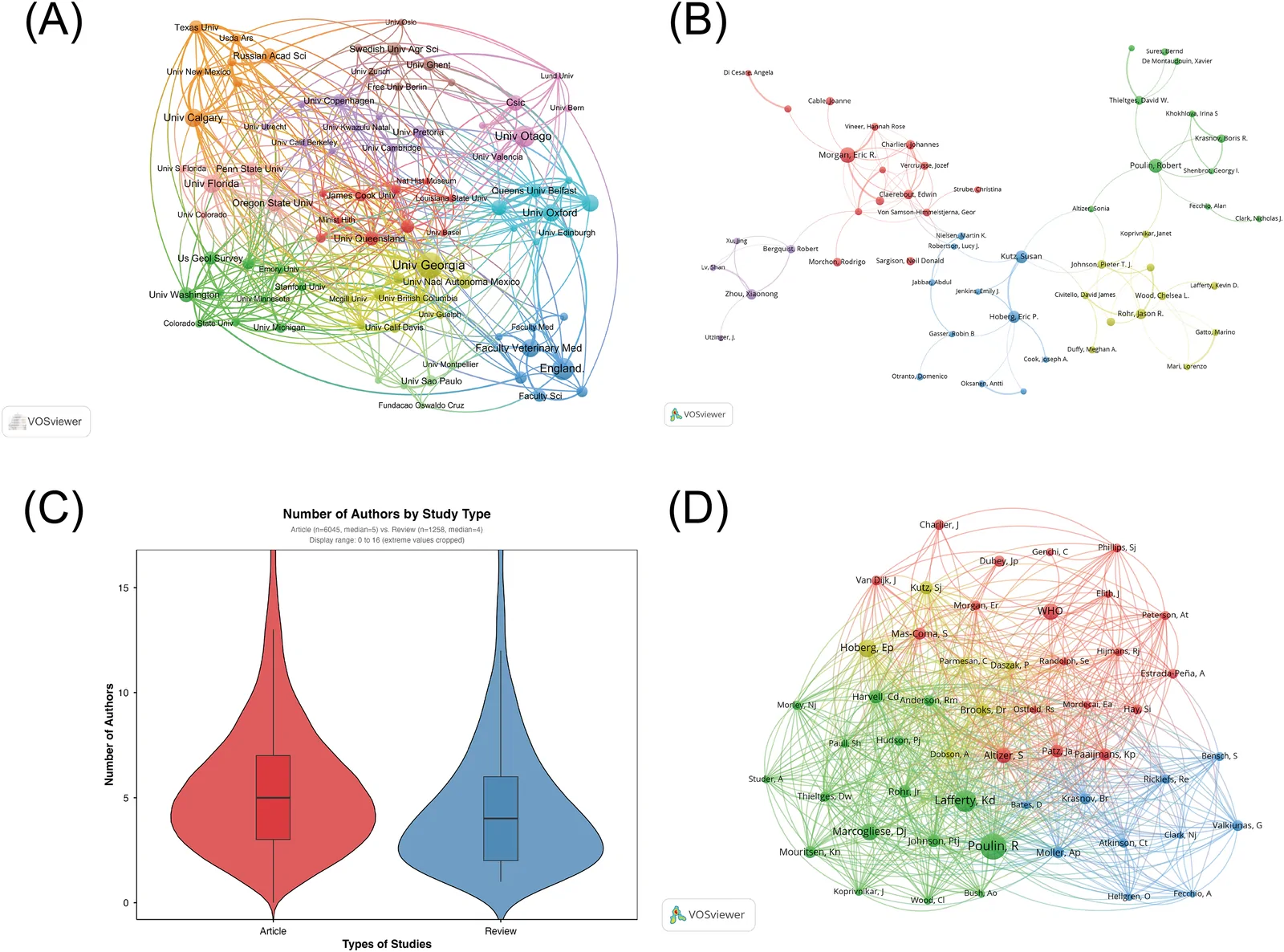
**(A)** Institutional collaboration network: Node size represents the research output level of the institution, and line thickness indicates the degree of collaboration between institutions. Thicker lines denote stronger collaborative relationships. **(B)** Author collaboration network: Node size reflects the research output level of authors, and line thickness represents the strength of collaboration between authors. Thicker lines indicate stronger collaborative ties. **(C)** Boxplot of the distribution of author counts across different research types, illustrating the difference in the number of authors between research articles and reviews, with extreme values excluded from the displayed range. **(D)** Author co-citation network: Node size represents the citation frequency of authors, while line thickness indicates the strength of co-citation between authors. Thicker lines signify a higher frequency of co-citation in the literature.

A total of 5,695 research institutions worldwide have participated in research in this field (**Table 3**). In terms of publication output, the French National Centre for Scientific Research (CNRS) ranks first with 229 publications, followed closely by the University of California System in the United States with 224 publications. Among the top ten institutions, seven are from the United States, two from France, and one from Spain, indicating the leading position of research institutions from the United States, France, and other countries in this field. From the perspective of institutional collaboration networks (**Figure 3A**), the University of Georgia in the United States and the University of Calgary in Canada exhibit the strongest connection strengths in the network (42 and 40, respectively), indicating that these two institutions play a pivotal role in promoting international academic collaboration. Other institutions, such as the University of Liverpool in the United Kingdom (39), the University of Florida in the United States (37), and the University of Bristol in the United Kingdom (33), also demonstrate strong international collaboration links. In the figure, node size represents the publication output of the institution, and line thickness represents collaboration strength. Overall, institutions from North America and Europe, represented by the United States, the United Kingdom, and Canada, form the core of the collaboration network, with U.S. institutions occupying a prominent position in both publication volume and network centrality.

**Table 3:** Top10 institutions related to climate and parasitic diseases research.

Rank	Institution	Publication	Original country/region	Rank	Institution	Total link strength	Original country/region
1	Centre National de la Recherche Scientifique(CNRS)	229	France	1	Univ Georgia	42	USA
2	University of California System	224	USA	2	Univ Calgary	40	Canada
3	University of Georgia	157	USA	3	Univ Liverpool	39	UK
4	University System of Georgia	151	USA	4	Univ Florida	37	USA
5	State University System of Florida	138	USA	5	Univ Bristol	33	UK
6	Consejo Superior de Investigaciones Cientificas(CSIC)	126	Spain	6	Univ Queensland	32	Australia
7	United States Department of the Interior	120	USA	7	Queens Univ Belfast	30	UK
8	United States Department of Agriculture(USDA)	113	USA	8	Texas Univ	28	USA
9	Pennsylvania State University	109	USA	9	Cornell Univ	28	USA
10	Inrae	107	France	10	Univ Oxford	26	UK

### 3.4 Distributions of authors and co-cited authors

A total of 33,078 authors have participated in research in this field, among whom 446 are core authors (with ≥5 publications), accounting for 1.35% of all authors but contributing 44.61% of the publications (3,258 articles), indicating a high concentration of research output (**Table 4**). In terms of individual productivity, the author with the highest number of publications is Professor Poulin, Robert from the University of Otago, New Zealand (51 articles), demonstrating outstanding performance in research sustainability. In terms of academic influence, the author with the highest citation count is Professor Johnson, Pieter T.J from the University of Colorado, United States (527 citations), whose research findings have garnered widespread attention and citations. Among the top ten authors with the highest publication output, four are from the United States, two from the United Kingdom, and one each from New Zealand, Canada, Spain, Cyprus, and Germany, highlighting the advantage of U.S. scholars in research productivity.

**Table 4:** Top10 authors in the field of climate change and parasites.

Rank	Author	Document	Country/ region	Institution	Rank	Author	Citation	Country/ region	Institution
1	Poulin, Robert	51	New Zealand	University of Otago	1	Johnson, Pieter T. J	527	USA	University of Colorado Boulder
2	Morgan, Eric R	50	UK	Queen’s University Belfast	2	Thomas, Matthew B.	491	USA	University of Georgia
3	Hoberg, Eric P	33	USA	University of New Mexico	3	Altizer,S	414	USA	University of Georgia
4	Kutz, Susan J	21	Canada	University of Calgary	4	Hoberg, Eric P	404	USA	University of New Mexico
5	Thomas, Matthew B	19	USA	University of Georgia	5	Paaijmans, Krijn P	371	South Africa	University of Witwatersrand
6	Johnson, Pieter T. J	18	USA	University of Colorado Boulder	6	Ward,JR	331	USA	Johns Hopkins University Applied Physics Laboratory
7	Morchon, Rodrigo	18	Spain	University of Salamanca	7	Rohr, Jason R	326	USA	University of Notre Dame
8	Rohr, Jason R	18	USA	University of Notre Dame	8	Paull,Sara H	302	USA	Battelle Memorial Institute - National Ecological Observatory Network, NEON
9	Karanis, Panagiotis	17	Cyprus	University of Nicosia	9	Dobson,AP	292	USA	Princeton University
10	Klimpel, Sven	17	Germany	Goethe University Frankfurt	10	Harvell,CD	292	USA	Cornell University

The author collaboration network analysis (**Figure 3B**) reveals the structure of scientific collaboration in this field. Five major collaborative groups can be identified in the network, each represented by nodes of different colors. Among them, the team centered around Morgan, E. R. (red cluster) exhibits the broadest external connections and plays a crucial bridging role in the collaboration network. In contrast, the teams led by Poulin, R. (green cluster) and Zhou, X. (deep purple cluster) are relatively independent and engage in fewer external collaborations. This pattern reflects the distinct roles and positioning of different research teams within the academic network. Furthermore, an analysis of author counts across different types of research literature (**Figure 3C**) shows a highly significant difference in the number of authors between research articles and review articles (*P* < 0.001). The median number of authors for research articles is 5(interquartile range, IQR:3–7), which is significantly higher than that for review articles, with a median of 4 authors (IQR:2–6) (**Figure 3C**).

Through the analysis of the author co-citation network (**Figure 3D**), four main knowledge communities within the field were identified, represented by Poulin, R (green), Moller, A. P. (blue), WHO (red), and Hoberg, E. P. (yellow) as core figures. These communities correspond to distinct theoretical foundations and research schools, and the high co-citation frequencies among authors within each group reflect close academic connections and strong communal influence. Notably, among the top ten co-cited authors, both WHO and Johnson, Ptj exhibit relatively high citation counts but comparatively low overall connection strength, indicating that the research topics or content they represent are broader in scope and their co-citation relationships with various academic clusters are more dispersed.

Over the past 25 years, the 7,303 publications in the field of climate and parasitic diseases have been widely distributed across 1,668 journals (averaging approximately 4 articles per journal). As shown in **Table 5**, *Veterinary Parasitology* (174 articles), *PLOS ONE* (166 articles), and *Parasitology* (142 articles) are the core journals with the highest publication volumes and citation frequencies. Notably, top-tier multidisciplinary journals such as *Science* (4,583 citations) and *Nature* (632 citations) have also published a considerable number of relevant studies, highlighting the importance and broad impact of research in this field. In terms of total link strength among journals, *Parasitology* (821), *International Journal for Parasitology* (718), and *Veterinary Parasitology* (600) rank in the top three, indicating that these journals occupy central positions in the knowledge dissemination network.

**Table 5:** Top10 journals related to climate change and parasites.

Rank	Journal	Publication	Rank	Journal	Citation	Rank	Journal	Total Link strength
1	Veterinary Parasitology	174	1	Veterinary Parasitology	6654	1	Parasitology	821
2	Plos One	166	2	Plos One	5178	2	International Journal for Parasitology	718
3	Parasitology	142	3	Parasitology	4608	3	Veterinary Parasitology	600
4	Parasitology Research	138	4	Science	4583	4	Global Change Biology	596
5	Parasites & Vectors	129	5	P NATL ACAD SCI USA	4101	5	Trends in Parasitology	516
6	International Journal for Parasitology	110	6	Nature	632	6	Plos One	464
7	Scientific Reports	105	7	International Journal for Parasitology	3575	7	Ecology Letters	439
8	Acta Tropica	96	8	P ROY SOC B-BIOL SCI	3533	8	Proceedings of the National Academy of Sciences of the United States of America	365
9	Plos Neglected Tropical Diseases	84	9	Parasites & Vectors	3517	9	Parasitology Research	352
10	Animals	82	10	J Parasitol	3443	10	Scientific Reports	337

**Figure 4:**
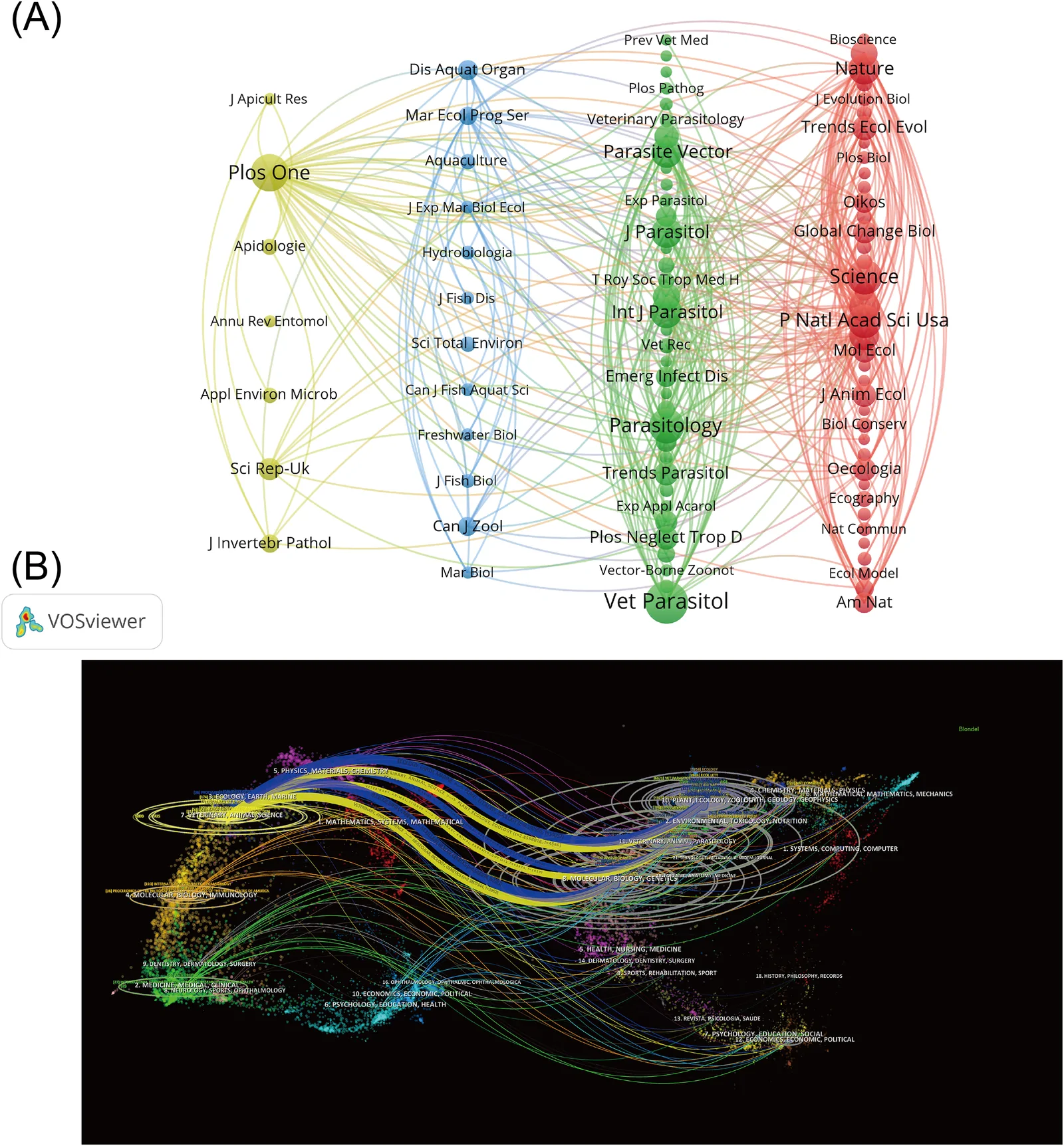
**(A)** This figure displays a journal co-citation network in the research field of climate change and parasitic diseases. Node size represents the citation frequency of the journals, and link thickness indicates the strength of co-citation between journals. **(B)** Dual-map overlay of publishing journals and cited journals. The left side shows the distribution of citing journals, and the right side shows the aggregation of cited journals. The curves connecting the two sides represent citation paths, with line width indicating the strength of the citation relationship. Thicker lines signify stronger citation ties.

Journal co-citation network analysis (**Figure 4A**) reveals knowledge linkages among different journals. Through clustering analysis, all journals are categorized into four primary knowledge clusters: the red cluster focuses on fundamental scientific research, including top-tier journals such as *Science*, *Nature*, and *PNAS*; the green cluster centers on parasitology research, featuring journals like *Veterinary Parasitology*, *Parasitology*, and *Parasites & Vectors*; the blue cluster primarily encompasses biology and zoology studies, including journals such as *Diseases of Aquatic Organisms* and *Marine Ecology Progress Series*; the yellow cluster leans toward entomology and pathology research. This journal clustering structure clearly reflects the interdisciplinary nature of the field.

The journal dual-map overlay analysis (**Figure 4B**) further reveals the knowledge flow paths between citing journals and cited journals. In the figure, the left side shows the distribution of citing journals, the right side shows the distribution of cited journals, and the curves connecting the two represent citation relationships. The analysis indicates that there are six main citation paths. Citing journals span multiple disciplinary fields, including medicine, psychology, education, ecology, biology, zoology, environmental science, genetics, mathematics, and systems science. This demonstrates that research findings in this direction are widely applied across numerous related disciplines, highlighting strong disciplinary penetration and the capacity for influence diffusion.

### 3.5 Distributions of reference

**Figure 5:**
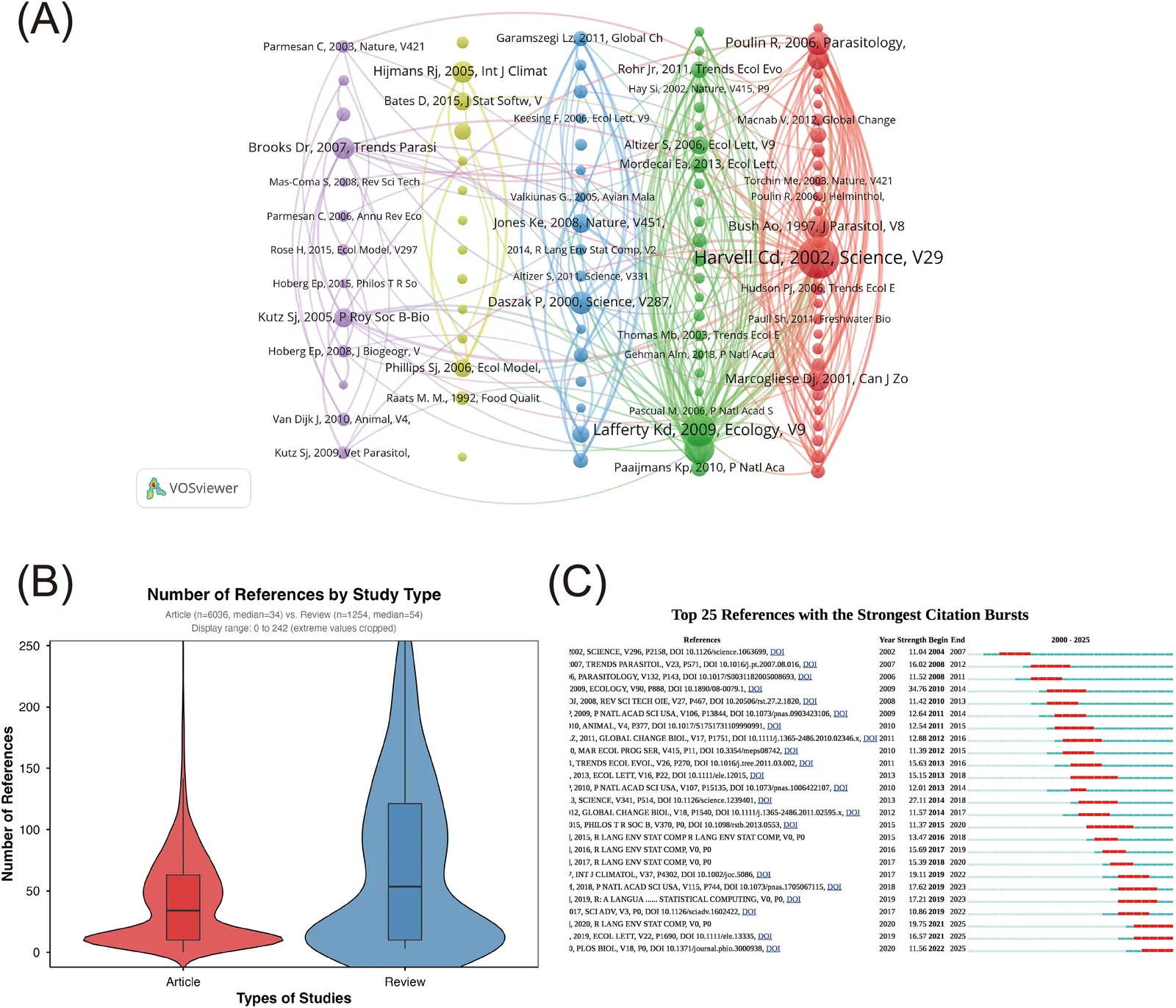
**(A)** Document co-citation network analysis in climate change and parasitic disease research. Node size represents the citation frequency of a publication, and line thickness indicates the strength of co-citation between publications. Thicker lines denote a higher frequency with which two publications are cited together in subsequent research. **(B)** Box plot of the distribution of reference counts across different research types, illustrating the difference in the number of references between research articles and reviews, with extreme values excluded from the displayed range. **(C)** Top 25 references with the strongest citation bursts(2000–2025). Blue segments indicate the overall time span of the study, while red segments mark the specific time periods during which a reference exhibit a citation burst.

The results of the annual high-citation literature analysis (**Table 6**) reveal the composition of the knowledge base in this field. The most influential publication is the review titled “Climate Warming and Disease Risks for Terrestrial and Marine Biota” by Harvell, CD et al., published in *Science*, with a cumulative citation count of 289. This article systematically explains how climate change threatens biodiversity through synergistic effects with infectious diseases. The second most cited is a study by Poulin, R et al. published in *Parasitology*, which experimentally demonstrated that rising temperatures significantly promote the release of cercariae in trematode parasites, with a total of 142 citations. This research indicates that climate warming not only affects the geographic distribution of parasites but may also exacerbate disease transmission risks by enhancing the proliferation of infectious stages. Other highly cited literature includes a review by Patz, JA et al. on the impact of environmental changes on emerging parasitic diseases (72 citations), a review by Marco Gliese, DJ et al. on the effects of climate change on parasitism in aquatic animals (135 citations), and a study by Kutz, SJ et al. on how global warming alters the dynamics of Arctic host-parasite systems (110 citations). These highly cited publications span multiple levels, from theoretical framework construction to specific mechanism exploration, collectively forming the core knowledge foundation of the field. The document co-citation analysis (**Figure 5A**) examines the frequency with which publications are cited together, revealing knowledge linkages among them. The analysis shows that publications such as Harvell CD (2002, *Science*) and Lafferty KD (2009, *Ecology*) are frequently co-cited in subsequent studies, indicating that they jointly constitute a widely recognized and valued knowledge base in the field of climate and parasitic diseases. The strong interconnections among these classic publications reflect the continuity and cumulative nature of research in this domain, providing a solid theoretical foundation and methodological guidance for subsequent studies.

**Table 6:** Top10 annual citation publication in the field of climate change and parasitic diseases.

Rank	First author	Year	Source	Article title	DOI	Citation	Document type
1	Patz, JA	2000	International Journal for parasitology	Effects of Environmental Change on Emerging Parasitic Diseases	10.1016/S0020-7519(00)00141-7	72	Review
2	Marcogliese, DJ	2001	Canadian Journal of Zoology	Implications of Climate Change for Parasitism of Animals in the Aquatic Environment	10.1139/cjz-79-8-1331	135	Review
3	Harvell, CD	2002	Science	Ecology-Climate Warming and Disease Risks for Terrestrial and Marine Biota	10.1126/science.1063699	289	Review
4	Kutz, SJ	2005	Proceedings of the Royal Society B-Biological Sciences	Global Warming Is Changing the Dynamics of Arctic Host-Parasite Systems	10.1098/rspb.2005.3285	110	Article
5	Poulin, R	2006	Parasitology	Global Warming and Temperature-Mediated Increases in Cercarial Emergence in Trematode Parasites	10.1017/S0031182005008693	142	Article
6	Altizer, S	2006	Ecology letters	Seasonality and the Dynamics of Infectious Diseases	10.1111/j.1461-0248.2005.00879	100	Review
7	Brooks, Daniel R	2007	Trends in Parasitology	How Will Global Climate Change Affect Parasite-Host Assemblages?	10.1016/j.pt.2007.08.016	128	Review
8	Dobson, Andy	2008	Proceedings of the National Academy of Sciences of the United States of America	Homage to Linnaeus: How Many Parasites? How Many Hosts?	10.1073/pnas.0803232105	71	Article
9	Marcogliese, D. J	2008	Revue Scientifique et Technique-Office International des Epizooties	The Impact of Climate Change on the Parasites and Infectious Diseases of Aquatic Animals	10.20506/rst.27.2.1820	126	Review
10	Paaijmans, Krijn P	2009	Proceedings of the National Academy of Sciences of the United States of America	Understanding the Link Between Malaria Risk and Climate	10.1073/pnas.0903423106	78	Article

The 7,303 publications included in this study (i.e., the citing literature) collectively referenced 241,558 citations, among which 73 references were co-cited 50 or more times. Details of the top 10 most-cited references are provided in **Table 7**. The most-cited reference is “Ecology-Climate Warming and Disease Risks for Terrestrial and Marine Biota,” followed by “The ecology of climate change and infectious diseases” and “Climate change and infectious diseases: from evidence to a predictive framework.” Notably, the distribution pattern and differences in the number of references cited show that review articles overall contain significantly more references than research articles (*P* < 0.001, **Figure 5B**), indicating that in the field of climate change and parasitic disease research, review literature is more likely to serve as core reference material. Reference burst refers to a period during which a particular reference is frequently cited. Using CiteSpace, burst-strength analysis was performed on the references, with the top 25 references in terms of burst strength shown in **Figure 5C**. The strongest burst comes from the paper published by Lafferty KD et al. in 2009 in *Ecology* (strength = 34.76, burst period 2010–2014), followed by the article by Altizer S et al. published in 2013 in *Science* (strength = 27.11, burst period 2014–2018).Overall, most high-burst references peaked between 2010 and 2020, and since 2015, new burst references have emerged each year, reflecting the ongoing evolution of the knowledge base and the continuous turnover of research hotspots in the field. Moreover, some references have maintained their burst status into 2025, indicating that they continue to exert considerable academic influence.

**Table 7:** Top 10 most cited references.

Rank	First author	Year	Source	Article title	DOI	Citation	Document type
1	Harvell, CD	2002	Science	Ecology-Climate Warming and Disease Risks for Terrestrial and Marine Biota	10.1126/science.1063699	289	Review
2	Kevin D Lafferty	2009	Ecology	The ecology of climate change and infectious diseases	10.1890/08-0079.1	213	Review
3	Sonia Altizer	2013	Science	Climate change and infectious diseases: from evidence to a predictive framework	10.1126/science.1239401	190	Review
4	R Poulin	2006	parasitology	Global warming and temperature-mediated increases in cercarial emergence in trematode parasites	10.1017/S0031182005008693	142	Article
5	P Daszak	2000	Science	Emerging infectious diseases of wildlife--threats to biodiversity and human health	10.1126/science.287.5452.443	135	Review
6	Marcogliese David J	2001	Canadian Journal of Zoology	Implications of climate change for parasitism of animals in the aquatic environment	10.1139/CJZ-79-8-1331	135	Review
7	Bush, A O	1997	Journal of Parasitology	Parasitology meets ecology on its own terms: Margolis et al. revisited	10.2307/3284227	134	Article
8	Daniel R Brooks	2007	Trends in Parasitology	How will global climate change affect parasite-host assemblages?	10.1016/J.PT.2007.08.016	128	Review
9	Hijmans, RJ	2005	International Journal of Climatology	Very high resolution interpolated climate surfaces for global land areas	10.1002/joc.1276	127	Article
10	Marcogliese, D. J	2008	Revue Scientifique et Technique-Office International des Epizooties	The impact of climate change on the parasites and infectious diseases of aquatic animals	10.20506/RST.27.2.1820	126	Review

**Figure 6:**
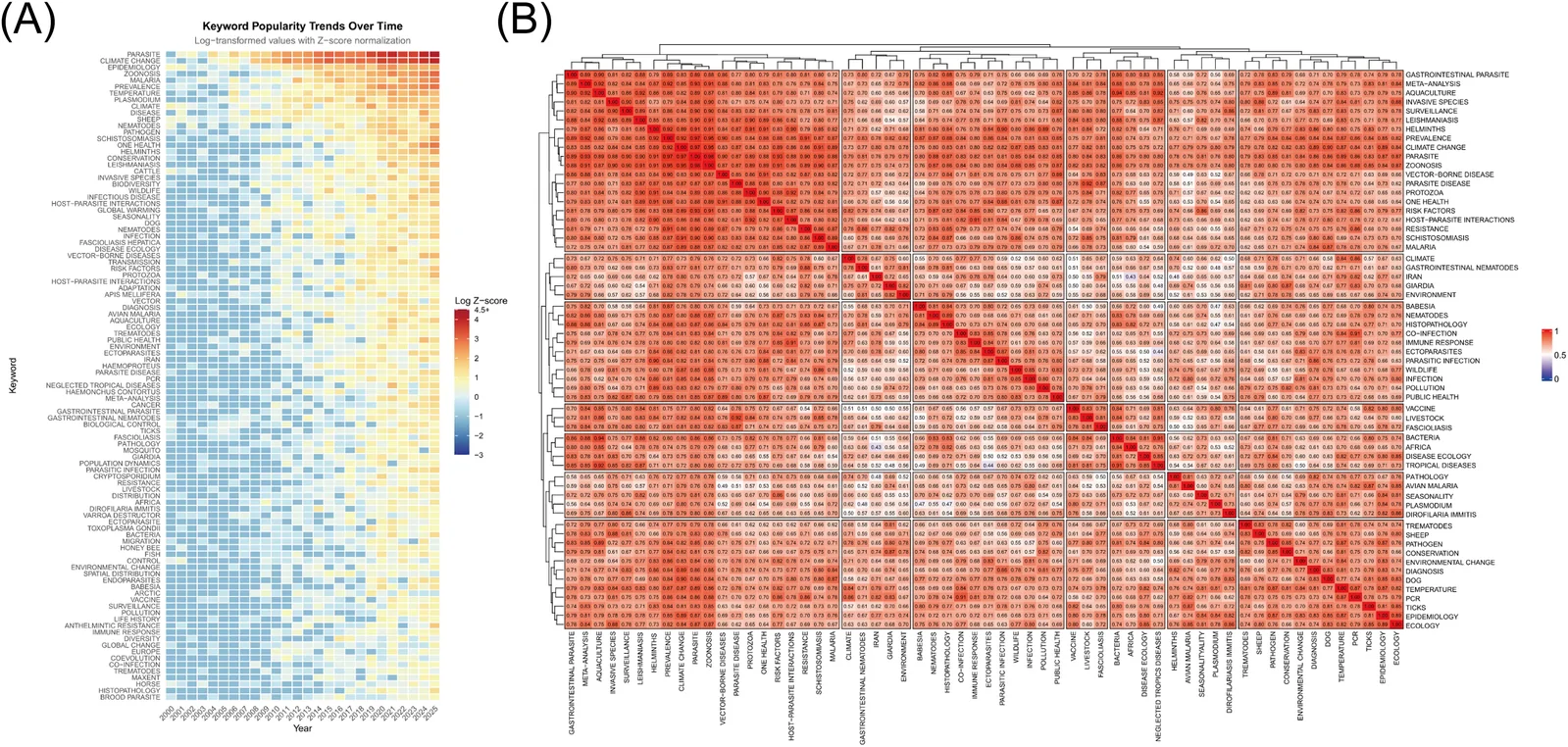
**(A)** Temporal evolution of keyword popularity in the field of climate change and parasitic disease research. The color intensity of each keyword in a given year represents its relative frequency compared to the total citation count of that year. **(B)** Correlation heatmap of keywords. Color depth indicates the strength of co-occurrence between keywords. Frequently co-occurring keywords are clustered and encoded with similar colors, reflecting the closeness of their thematic associations.

### 3.6 Keyword analysis

**Figure 7:**
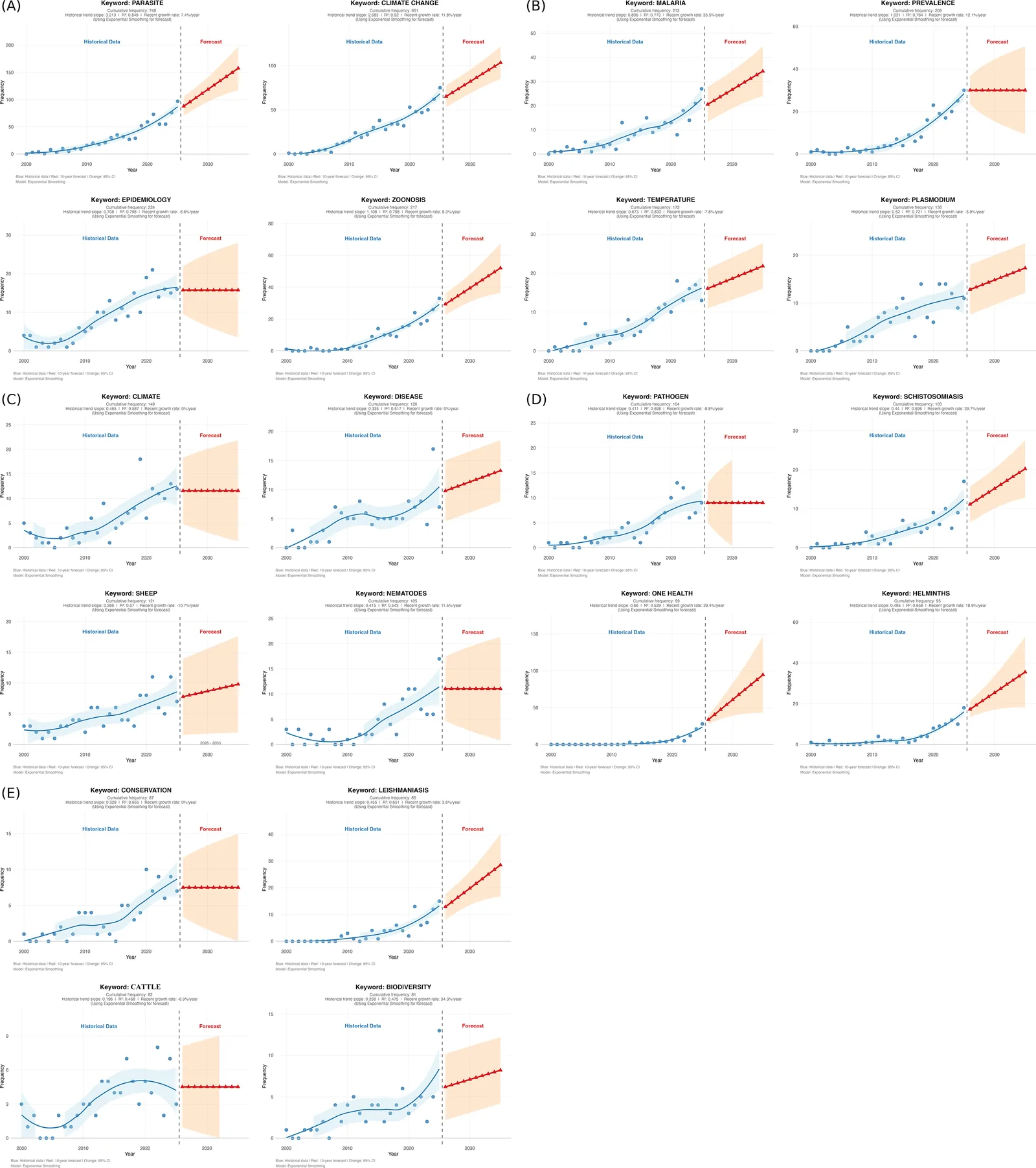
The exponential smoothing forecast of the cumulative publication volume for five core keyword groups (**(A)** Parasite, Climate Change, Epidemiology, Zoonosis; **(B)** Malaria, Prevalence, Temperature, Plasmodium; **(C)** Climate, Disease, Sheep, Nematodes; **(D)** Pathogen, Schistosomiasis, One Health, Helminths; **(E)** Conservation, Leishmaniasis, Castle, Biodiversity) during 2000–2030, where the blue line represents historical data, the red line indicates the forecast for the next 10 years, and the orange shaded area denotes the 95% confidence interval. This extrapolation is an exploratory tool to visualize historical growth patterns and should not be interpreted as a precise quantitative prediction of future research output.

This study comprehensively applies time-trend analysis, clustering and network structure analysis, and future trend prediction to conduct a multidimensional bibliometric investigation of core keywords in the field of climate change and parasitic disease research from 2000 to 2025, systematically revealing the dynamically evolving knowledge structure, research hotspots, and development directions of this field. Three heatmaps provide complementary perspectives from the dimensions of historical evolution (**Figure 6A**), clustering structure (**Figure 6B**), and future trends (**Figure 7**).

#### 3.6.1 Temporal evolution and overall development trends

The temporal trend analysis of keywords (**Figure 6A**) indicates that the research focus remains firmly anchored on two foundational themes, “Parasite” and “Climate change,” which together form the intellectual backbone of the field. Meanwhile, the research frontier is expanding rapidly. Emerging directions typified by “One health,” “Leishmaniasis,” and “Surveillance” show remarkable vitality (with some growth rates exceeding 200%), marking a deepening shift toward interdisciplinary integration and early warning systems for emerging infectious diseases. Different themes exhibit distinct development patterns: some traditional research areas (for example, those related to “Sheep”) demonstrate relatively high annual stability, while high-growth hotspots (for example, “One health”) are in a phase of rapid exploration with substantial year-to-year fluctuations. Overall, the vast majority of themes are in stages of “rapid growth” or “steady development,” driving the field from traditional epidemiological investigations toward an increasingly integrated framework that encompasses climate modeling, biodiversity conservation, and global health governance.

#### 3.6.2 Clustering structure and core research themes

Through hierarchical cluster analysis of the correlation matrix of core keywords, this study identifies seven highly cohesive thematic groups in this field (**Figure 6B**, **Table 8**), with an average correlation coefficient of 0.705 and 198 strong correlation pairs (r > 0.8), confirming the significant clustering of research hotspots. The specific clustering results are shown in **Table 8**. Among these, the “Core Research on Parasitic Diseases” cluster is the largest and has the strongest internal associations (average r=0.845). Centered on “Parasite,” it forms a dense network of strong connections with keywords such as “Zoonosis,” “Prevalence,” and “Climate change,” thus laying the research foundation for the entire field. The “Climate Epidemiology” cluster highlights the close integration of climatic variables (for example, “Temperature”) with epidemiological methods (“Epidemiology”). Importance analysis shows that the top five keywords (Parasite, Climate change, Zoonosis, Prevalence, Helminths) all belong to the core cluster, further confirming the central position of this group.

**Table 8:** Keyword clustering analysis in the field of parasitic diseases and climate change research.

Cluster ID	Number of keywords	Internal average correlation	Thematic classification	Representative keywords
1	20	0.845	Core Research on Parasitic Diseases	Parasite, Zoonosis, Prevalence
2	12	0.769	Climate Epidemiology	Temperature, Epidemiology, Pathogen
3	11	0.735	Parasite Infection Mechanisms	Ectoparasites, Parasitic Infection, Infection
4	4	0.814	Ecology of Bacteria and Diseases	Bacteria, Neglected Tropical Diseases, Disease Ecology
5	3	0.808	Parasite Control in Animal Husbandry	Livestock, Vaccine, Fascioliasis
6	5	0.718	Environment and Parasite Transmission	Giardia, Gastrointestinal Nematodes, Environment
7	5	0.697	Special Parasitic Disease Research	Plasmodium, Helminths, Avian Malaria

#### 3.6.3 Strong−correlation network and distribution of research hotspots

The strong-correlation network (r > 0.8) precisely identifies the current core research combinations (**Figure 6B**). The most prominent relationships include: the strong association between “Parasite” and “Zoonosis” (r = 0.983) as well as “Climate change” (r = 0.965), clarifying the core theme of “climate-change-driven zoonotic parasitic diseases”; the close link between “Parasite” and “Prevalence” (r = 0.967) highlights the importance of infection rate as a key quantitative indicator. Based on clustering and strong-correlation analysis, this study identifies five highly co-occurring keyword clusters with strong internal correlations (**Figure 6B**), representing consolidated thematic areas in the field:①Mechanisms of climate change impacts on parasite transmission;② Epidemiological characteristics of zoonotic parasitic diseases;③ Interdisciplinary application of methods such as Meta-Analysis, Diagnosis, and PCR;④Climate responses of specific diseases including Schistosomiasis, Leishmaniasis, and Malaria;⑤Response strategies encompassing One Health, Public Health, and Surveillance. Detailed clustering information and correlation data for the relevant heatmaps are provided in the **Supplementary Materials**.

#### 3.6.4 Development trends and future prospects

Trend prediction analysis (**Figure 7**) further validates and deepens the above findings. The forecast and 95% confidence interval are based on exponential smoothing of historical data (blue line). This extrapolation is an exploratory tool to visualize historical growth patterns and should not be interpreted as a precise quantitative prediction of future research output. “Parasite” and “Climate change,” with cumulative frequencies of 748 and 651 respectively and significant growth trends (R² = 0.849 and 0.920), solidly maintain their dominant positions. The continued growth of keywords such as “Epidemiology,” “Zoonosis,” and “Malaria” collectively constructs a multidimensional research framework centered on parasites, set against the backdrop of climate change and employing epidemiological methodologies. Particularly noteworthy is the strong upward momentum of comprehensive concepts such as “One Health” and “Biodiversity” (**Figure 7**). Their growth slopes and expanding association networks suggest a profound shift in the research paradigm—from a singular focus on disease control toward an integrated framework that encompasses ecosystem health, biodiversity conservation, and global health governance. The top 20 high-frequency keywords account for nearly half of the total occurrence frequency, and the majority show significant growth, indicating that this interdisciplinary field is well-grounded and dynamic. It holds promising potential for continued deepening and expansion into broader dimensions such as environmental health and system resilience in the future.

## 4 Discussion

It is important to note that the patterns identified here are descriptive in nature, reflecting trends in publication activity and thematic clustering. While they offer insights into the development of the field, they should not be interpreted as direct evidence of causal relationships or as precise forecasts of future research directions.

### 4.1 General trends

Through a bibliometric analysis of the literature in the field of climate change and parasitic diseases, this study comprehensively reveals the developmental dynamics and knowledge structure of this research area. The results show that the annual number of publications in this field has exhibited a significant upward trend, with growth accelerating notably after 2015. The publication output in 2025 increased by approximately 2070.6% compared to that in 2005, and from 2001 to 2024, it maintained a growth rate of around 1545.0%. This indicates that research on climate change and parasitic diseases has attracted increasing attention and scholarly effort.

At the national/regional level, the global research landscape exhibits marked disparities. The United States holds an overwhelmingly dominant position in this field, leading significantly in terms of total publication output, total citations, and corresponding author contributions. Its leading advantage stems from sustained and stable investment in research, a well−established academic system, and a robust international collaboration network. Developed European countries (such as the United Kingdom, Germany, and France) also demonstrate strong research capabilities and have formed a close core network of scientific collaboration with the United States, jointly driving knowledge production in this field. Notably, contributions from developing countries such as China (1,148 publications, ranking 5th) and Brazil (1,035 publications) are becoming increasingly substantial. As early as 2000, Githeko et al. highlighted in the *Bulletin of the World Health Organization* the impact of climate change on malaria transmission in Africa, noting that developing countries are both highly vulnerable to climate change impacts and crucial bases for related research (Githeko et al., 2000). However, in terms of average citations per article (China averages 4.9 citations per article, while the U.S. averages 10.9), developed countries still maintain an edge in research quality and influence, suggesting that Global South nations need to continue efforts to enhance the quality of their research. Therefore, from the perspective of reducing national health risks and addressing practical needs, developing countries should particularly increase investment in scientific research, cultivate local expertise, actively deepen international cooperation, and integrate into global research networks to build effective public health defenses.

At the institutional level, approximately 70% of the top ten institutions by publication output are based in the United States, with the remainder located in France (n=2) and Spain (n=1), further reinforcing the above-described pattern. In contrast, although research institutions from developing countries have increased in number, there remains considerable room for improvement in terms of academic influence. This uneven distribution may affect the diversification of research agendas and lead to insufficient attention to certain important regions or topics. Therefore, we should advocate for and promote a more inclusive and diversified global research landscape. Efforts should be made to encourage and support research institutions from more regions—particularly those in high−disease−burden countries—to deeply integrate into international knowledge networks, working together to address global health challenges.

Among the top ten most productive journals, the vast majority are specialized publications in veterinary or classical parasitology (such as *Veterinary Parasitology*, *Parasitology*, *International Journal for Parasitology*, etc.). Among these, *Veterinary Parasitology* and *Parasitology* rank at the forefront in terms of both publication volume and citation counts, and can be regarded as the “core platforms” of this interdisciplinary field. At the same time, top-tier comprehensive journals such as *Science* and *Nature* have also published several highly influential papers, demonstrating that these academic journals play a crucial role in advancing research in this area. A notable feature of the journal distribution is the prominence of veterinary and ecological parasitology journals (e.g., *Veterinary Parasitology*, *International Journal for Parasitology*) alongside clinical infectious disease journals. This reflects the interdisciplinary nature of climate−change research on parasites, which often spans wildlife, livestock, and human health.

Author analysis reveals a nonlinear relationship between academic influence and publication quantity. For example, Poulin, Robert from the University of Otago, New Zealand, ranks first with 51 publications, while Johnson, Pieter T. J from the University of Colorado, United States, has published only 18 articles yet leads in citations with 527. This indicates that research quality, innovation, and the significance of the addressed problems are key determinants of academic impact. Collaboration network analysis further shows that authors have formed several closely connected research clusters. Highly influential scholars such as Johnson, Pieter T. J belong to a highly collaborative subgroup along with Rohr, Jason R and Civitello, David J., reflecting the concentration of research directions and the importance of teamwork. While authors from the United States dominate the core author group, scholars from countries such as New Zealand, the United Kingdom, and Canada also feature prominently through high−quality research, demonstrating that the field maintains a degree of international openness alongside its core dominance.

The results of this study indicate that the knowledge system in the field of climate change and parasitic diseases exhibits a distinct “review−dominated” characteristic. Analyses show that review articles are cited significantly more frequently than original research papers and occupy a central position in both high−citation and co−citation networks. This phenomenon may be related to the disciplinary nature of the field: it integrates knowledge from multiple domains such as ecology, climate science, and medicine, requiring researchers to systematically grasp cross−disciplinary advances through review literature. This differs from fields where experimental techniques evolve rapidly and where citing the latest primary data is often prioritized. Furthermore, reference burst analysis reveals that the period from 2010 to 2020 represents the main phase during which key literature was intensively cited. This timeframe broadly coincides with the release of the IPCC assessment reports and the promotion of the “One Health” concept, suggesting that the development of the field has been driven by macro−level scientific consensus. The continued burst activity of some recent literature indicates that related topics remain under active scholarly attention and that research in this area is still deepening.

Overall, the field of climate change and parasitic disease research is in a phase of rapid development, and sustained efforts are needed in the following areas: first, to further enhance research quality, particularly by deepening the exploration of the complex mechanisms linking climate change and parasitic diseases; second, to promote a more balanced allocation of global scientific resources and support capacity−building in developing countries; third, to strengthen interdisciplinary collaboration and exchange to foster innovation in research paradigms; and fourth, to improve the translation of research findings into policy, providing a scientific basis for global public health governance. Only through the collective efforts of the global academic community can we better address the health challenges posed by climate change and contribute to building a global community of health for all.

**Figure 8:**
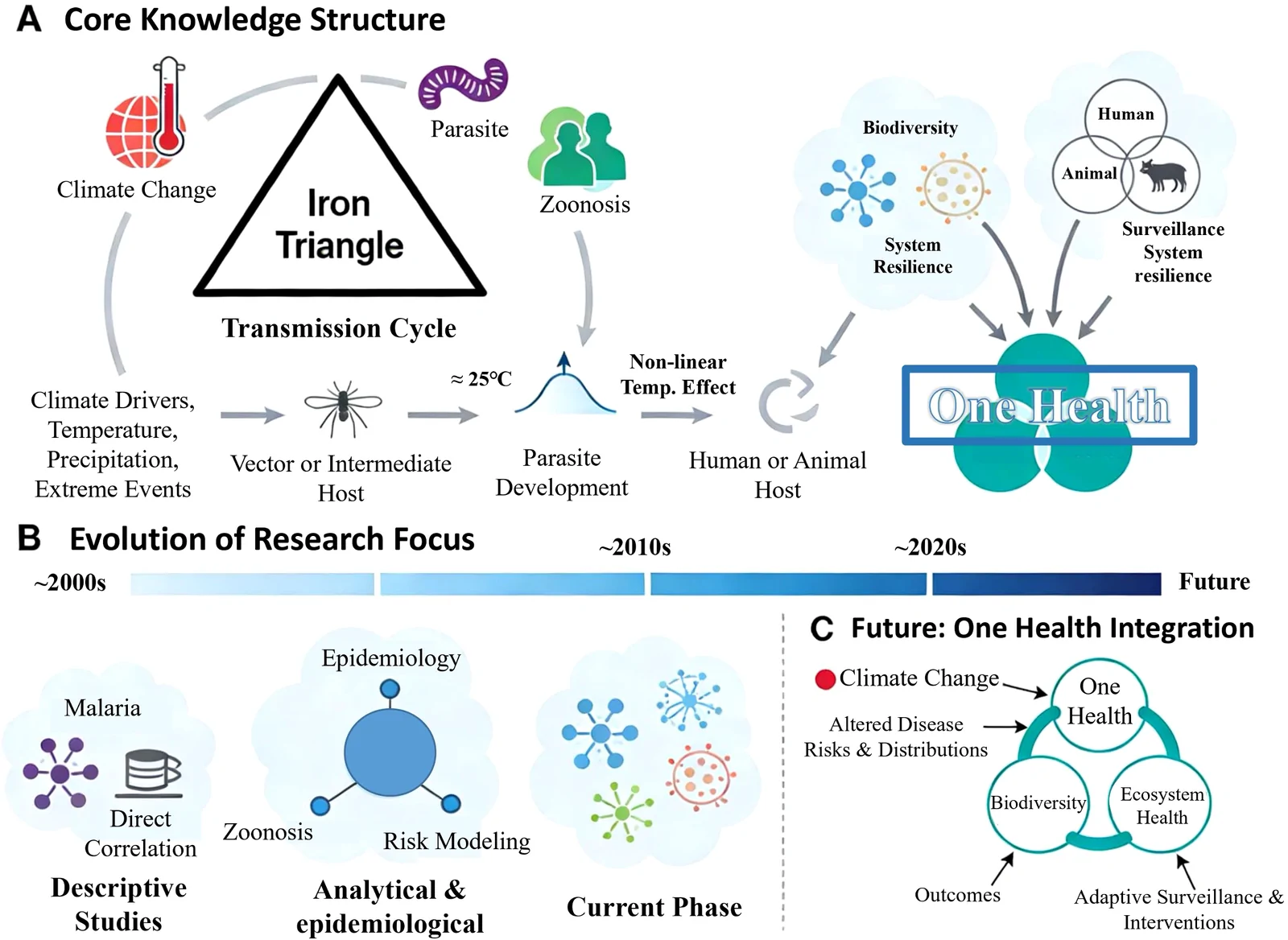
Conceptual evolution of climate change and parasitic disease research. **(A)** Core Structure. The foundational “Iron Triangle” links Climate Change, Parasites, and Zoonosis, underpinned by a mechanistic transmission cycle influenced by key climate drivers. **(B)** Thematic Progression. The field has evolved from early descriptive studies (∼2000s), through analytical and epidemiological phases (∼2010s), toward integration within a One Health framework (∼2020s–Present). **(C)** Future Integration. The One Health paradigm synthesizes climate-driven risks and biodiversity considerations to guide adaptive surveillance and interventions.

The whole conceptual evolution of climate change and parasitic disease research is shown in **Figure 8**.

### 4.2 Research hotspots and development trends

The analysis of the results of this study indicates that the research field of climate change and parasitic diseases has formed a knowledge system characterized by clear structure, solid framework, and dynamic development. Its evolutionary trajectory reflects a developmental path from foundational to cutting−edge, from singular to multiple perspectives, and from descriptive to integrative approaches. Current research centers on the interaction between Climate change and Zoonosis, forming highly clustered thematic networks. These clusters are rapidly integrating with interdisciplinary paradigms such as One Health, demonstrating strong vitality and broad prospects for future development.

#### 4.2.1 Evolution and maturity of the field: from isolated breakthroughs to systemic integration

Analysis indicates that this field has evolved from an incipient stage focused on relationships between specific parasites and climate variables into a mature, highly active interdisciplinary discipline with a solid foundation. Its evolutionary pathway exhibits clear phases: the early period (early 2000s) was dominated by empirical studies describing the effects of Climate change on traditional diseases such as Malaria; the middle period (2010s) saw the growing prominence of Zoonosis and Epidemiology methods, with research networks becoming increasingly cohesive; the recent period (2020s) reveals a strong trend toward integration into systemic frameworks such as One Health, Biodiversity, and Surveillance. This evolution is reflected not only in the exponential growth of literature but, more importantly, in a shift in research paradigms—from single−etiology analyses to systemic exploration of the complex interactions at the “environment–animal–human” health interface. Currently, the vast majority of themes are in stages of “rapid growth” or “steady development,” confirming the field’s robust and sustained research momentum.

#### 4.2.2 Stability and internal heterogeneity of the core knowledge structure

This study reveals that the field has formed a tightly interlinked knowledge network centered on the “Parasite−Climate change−Zoonosis” triad. Parasite, as the research object, together with Climate change (the environmental driver) and Epidemiology (the methodological approach), constitutes the most fundamental and stable conceptual framework of the discipline. The exceptionally high internal correlation (0.845) and the large number of strong linkage pairs within Cluster 1 (Core Research on Parasitic Diseases) indicate that the transmission mechanisms, epidemiological characteristics, and risks of zoonotic parasitic diseases under climate change have become a well−established, highly productive core research direction. However, significant heterogeneity exists within the knowledge network. Clusters that intersect deeply with environmental science and ecology (e.g., Cluster 6, “Environment and Parasite Transmission”) and those focusing on specific parasitic diseases (e.g., Cluster 7, “Special Parasitic Disease Research”) show relatively lower internal correlations (0.718 and 0.697, respectively), suggesting that the knowledge systems in these sub−fields remain loosely structured and contain research gaps. For instance, quantitative and mechanistic studies on how environmental factors (e.g., precipitation patterns, extreme weather events) influence parasite transmission are still insufficient; understanding of climate responses in specific parasitic diseases such as Avian malaria remains limited; and research on climate−adaptive interventions (e.g., vaccine efficacy evaluation) is relatively weak. This heterogeneity suggests that while consolidating core themes, future efforts should focus on strengthening these underdeveloped yet critical interdisciplinary directions.

#### 4.2.3 External drivers and field development responses

The dynamic trends of keywords clearly reflect the field’s response to major global scientific agendas and societal events. The accelerated growth of multiple keywords around 2015 closely aligns with the surge in global attention to climate change following the signing of the Paris Agreement, indicating that the research agenda of this field is highly synchronized with international political consensus. Meanwhile, short−term fluctuations in certain keywords (e.g., Epidemiology) during 2020–2022 likely stem from the temporary reshaping of global research resources and focus due to the COVID−19 pandemic. The imprint of these external “signals” in bibliometric data demonstrates that this field is not a closed academic system but rather a dynamic research domain that actively engages with real−world challenges.

#### 4.2.4 Research progress on climate responses of key parasitic diseases

Malaria, as the most climate−sensitive parasitic disease, has been studied most systematically and in−depth. Early research primarily focused on the direct effects of temperature on malaria transmission, whereas recent studies have adopted more diverse perspectives. Research by Paaijmans et al. revealed that daily temperature fluctuations exert a greater influence on malaria transmission than mean temperature: under the same average temperature, larger diurnal temperature variations significantly inhibit Plasmodium development within mosquitoes (Paaijmans et al., 2010). This finding has prompted researchers to reconsider the selection of temperature indicators and to incorporate temperature variability into subsequent models. Changes in spatial distribution represent another important research direction. Using historical climate data and malaria incidence records combined with future climate scenarios, Ryan et al. projected that by 2050, areas suitable for malaria transmission may expand toward higher altitudes, exposing previously low−risk regions to new threats (Ryan et al., 2020). This study specifically highlighted the vulnerability of highland areas in Africa, providing a scientific basis for targeted control strategies. Beyond malaria, climate response research on other parasitic diseases is also advancing. Studies on the climate response of schistosomiasis have deepened. Zhou et al. developed a climate−ecological model for schistosomiasis transmission in China, predicting that under moderate emission scenarios, the suitable transmission area for schistosomiasis may expand by 12% by 2050,with the transmission season potentially extending by 1–2 months (Zhou et al., 2008). This research integrated not only temperature effects but also hydrological changes, land use, and socioeconomic factors, reflecting a multifactorial analytical approach. Furthermore, Yang et al. analyzed the impact of precipitation pattern changes on schistosomiasis transmission in China, finding that flooding following heavy precipitation events may facilitate the dispersal of intermediate host snails to new water bodies, thereby increasing transmission risk (Yang et al., 2005). Leishmaniasis, as a neglected tropical disease, is gradually gaining attention regarding its climate change response. A systematic review by Petersen et al. noted that rising temperatures may promote the northward expansion of sand fly vector distributions while shortening the development time of pathogens within sand flies, potentially increasing transmission risk (Paez-Espinosa et al., 2025). However, such studies face challenges due to data scarcity, particularly in regions with weak surveillance systems. Using ecological niche models, González et al. projected distribution shifts of cutaneous leishmaniasis in the Americas under climate change, suggesting that suitable transmission areas may move toward higher altitudes and latitudes (Gonzalez et al., 2014). Climate response research on other parasitic diseases is also progressively being conducted. For hookworm, Brooker et al. (2006) analyzed the effects of climate change on soil−transmitted helminthiases, noting that temperature increases may accelerate the development of hookworm eggs in soil but could also reduce soil moisture, leading to complex combined effects (Brooker et al., 2006). Regarding echinococcosis, Atkinson et al. examined the impact of climate change on the life cycle of Echinococcus granulosus, finding that higher temperatures may shorten the survival time of eggs in the environment but could also extend the grazing season of intermediate host sheep, thereby increasing infection opportunities (Atkinson et al., 2013).

#### 4.2.5 In−depth analysis of climate impacts on parasite transmission mechanisms

Early studies primarily focused on statistical associations between climatic variables such as temperature and precipitation and the incidence of parasitic diseases, whereas recent research has delved into specific physiological and ecological mechanisms (Wolmuth-Gordon et al., 2025). Temperature affects multiple stages of parasite life cycles, including development rates, reproductive capacity, vector behavior, and host susceptibility. A classic study by the Poulin team systematically examined the effect of temperature on cercarial release in trematode parasites under controlled laboratory conditions, finding that each 1 °C increase in temperature raised cercarial release by 8.5%, with this effect being more pronounced at lower latitudes (Poulin, 2006). This research not only quantified the temperature effect but also suggested that climate warming could exacerbate disease transmission risk by enhancing the proliferation of infectious stages. Similarly, Mordecai et al. developed a mathematical model linking temperature to Plasmodium development rates and mosquito biting rates, revealing that the optimal temperature for malaria transmission is approximately 25 °C, with transmission efficiency declining both above and below this threshold. This nonlinear relationship holds significant implications for predicting disease risks under climate change (Mordecai et al., 2013). Changes in precipitation patterns also receive broad attention regarding their impact on parasite transmission. Stensgaard et al. investigated the influence of precipitation variation on schistosomiasis transmission in Africa, showing that seasonal rainfall not only affects the abundance of snail intermediate hosts but also alters human water-contact frequency, thereby influencing infection risk (Stensgaard et al., 2019). Extreme precipitation events may expand the distribution range of snails through flooding while increasing human exposure to infested water, elevating the risk of schistosomiasis outbreaks (Ayob et al., 2023).

#### 4.2.6 Future prospects: toward one health and system resilience

Based on the observed growth trajectories of emerging keywords, we tentatively suggest that the field may be advancing toward the following directions, although such trend-based extrapolations should be viewed as speculative and subject to various uncertainties. First, predicting the geographic shifts of pathogens and hosts driven by Climate change will remain a key focus, requiring higher-resolution models and better integration of empirical data. Second, research on integrated prevention and control strategies guided by the One Health framework will occupy a more central position, aiming to synergistically advance human health, animal health, and ecosystem health. Finally, the synergy between Biodiversity conservation and disease control will emerge as a new frontier, exploring how biodiversity loss may alter host community structure and thereby influence disease risk.

## 5 Limitation

This study systematically maps the developmental trajectory and knowledge structure of the field of climate change and parasitic diseases using bibliometric methods, yet several limitations should be noted. First, although two major databases—Web of Science and Scopus—were integrated, some regional literature may have been omitted. Moreover, the literature types were restricted to academic articles and reviews, excluding conference proceedings and monographs, which may limit the comprehensiveness of the field portrait. Second, recently published literature may not have been fully captured in terms of academic impact due to shorter citation cycles. Thirdly, the broad search strategy, while enabling a comprehensive mapping of the interdisciplinary field, also means the dataset extends beyond strictly clinical human parasitology. This should be considered when interpreting the findings from a purely medical perspective. Moreover, The inclusion of a substantial number of veterinary and ecological studies may limit direct comparability with purely clinical parasitology literature; however, it enables a holistic mapping of the interdisciplinary field aligned with One Health principles.

Additionally, inherent variations in literature quality could affect the reliability of the analysis. To address these limitations, future research plans to expand literature sources by incorporating specialized and regional databases such as PubMed and CNKI, and to diversify document types by including high-quality conference papers and book chapters. Furthermore, stratified analysis based on journal impact, document type, and peer-review recognition will be conducted to mitigate quality-related biases. These steps aim to enhance the representativeness and reliability of the findings, providing a more accurate reflection of the current research landscape and future trends in this field.

## 6 Conclusion

This study employs bibliometric analysis to review trends, hotspots, and frontiers in research on climate change and parasites from January 1, 2000 to December 31, 2025. Visual analyses indicate that the field is in a phase of rapid development, with a steady growth in publications and continuous emergence of new research. Prominent contributors such as Poulin, Robert and Johnson, Pieter T. J. have significantly advanced the field. The United States leads in the number of published papers, reflecting its active engagement in this research area. These findings underscore the importance of further research on climate change and parasites, as well as the potential for continued collaboration among regions including North America and Europe, North America and Asia, within Oceania and Europe, and within Asia itself. Journals such as *Veterinary Parasitology* demonstrate higher publication volumes and citation frequencies, playing a key role in disseminating research and driving knowledge progress in the field. Based on the observed trends, future research is likely to focus on developing high-resolution models to accurately predict climate-driven changes in disease distribution, promoting integrated prevention and control through the One Health framework, and deeply exploring the synergistic relationship between biodiversity conservation and disease risk control as an emerging frontier. Overall, this study provides valuable insights into research trends and themes related to climate change and parasites, paving the way for further exploration and collaboration in this critical research area.

## Data Availability

The original contributions presented in the study are included in the article/**Supplementary Material**. Further inquiries can be directed to the corresponding author.
