# Correction: Global research trends at the intersection of climate change and parasitic diseases: a systematic bibliometric research (2000–2025)

**DOI:** 10.3389/fcimb.2026.1937014

**Published:** 2026-08-10

**Authors:** Maireyamuguli Nuermaimaiti, Zile Liu, Yimei Xu, Xinwei Qi

**Affiliations:** 1 Xinjiang Medical University First Affiliated Hospital Medical Laboratory Center, Urumqi, China; 2 School of Public Health, Xinjiang Medical University, Urumqi, China; 3 College of Anesthesiology, Southern Medical University, Guangzhou, China; 4 The First Affiliated Medical College, Southern Medical University, Guangzhou, China; 5 Xinjiang Uyghur Autonomous Region Center for Disease Control and Prevention, Urumqi, China

**Keywords:** bibliometric studies, climate change, one health, parasitic diseases, visual analysis

Author Yimei Xu was omitted as an author in the published article. The correct author list reads: “Maireyamuguli Nuermaimaiti, Zile Liu, Yimei Xu, and Xinwei Qi”

Author Yimei Xu was erroneously omitted as corresponding author. The correct corresponding authors are: Yimei Xu, 545763836@qq.com; Xinwei Qi, qxww2008@126.com.

The **Author contributions** Statement has been corrected to read:

“MN: Conceptualization, Data curation, Formal analysis, Investigation, Methodology, Validation, Visualization, Writing – original draft, Writing – review & editing. ZL: Conceptualization, Data curation, Formal analysis, Methodology, Software, Validation, Visualization, Writing – original draft, Writing – review & editing. YX: Writing - review and editing, Supervision, Formal analysis. XQ: Data curation, Formal analysis, Investigation, Methodology, Supervision, Validation, Visualization, Writing – review & editing”.

Author Maireyamuguli Nuermaimaiti was erroneously spelled as “Nuermaimaiti Maireyamuguli”.

The original version of this article has been updated.

